# Phosphodiesterase 4D polymorphisms associate with the short-term outcome in ischemic stroke

**DOI:** 10.1038/srep42914

**Published:** 2017-02-22

**Authors:** Yan-li Song, Chun-juan Wang, Yi-ping Wu, Jie Lin, Peng-lian Wang, Wan-liang Du, Li Liu, Jin-xi Lin, Yi-long Wang, Yong-jun Wang, Gai-fen Liu

**Affiliations:** 1 Department of Neurology, Beijing Tiantan Hospital, Capital Medical University, Beijing, China; 2 China National Clinical Research Center for Neurological Diseases, Beijing, China; 3 Center of Stroke, Beijing Institute for Brain Disorders, Beijing, China; 4 Beijing Key Laboratory of Translational Medicine for Cerebrovascular Disease, Beijing, China; 5 Department of Neurology, Handan First Hospital, Handan, China.

## Abstract

It has been demonstrated that phosphodiesterase 4D (PDE4D) genetic polymorphism is associated with ischemic stroke. However, the association between PDE4D gene and prognosis after ischemic stroke remains unknown. We consecutively enrolled ischemic stroke patients admitted to Beijing Tiantan Hospital from October 2009 to December 2013. Clinical, laboratory and imaging data upon admission were collected. All patients were followed up 3 months after stroke onset. Odds ratios (ORs) and 95% confidence intervals (CIs) were calculated to assess the associations of genetic polymorphisms with 3-month outcome after ischemic stroke and different subtypes, under various genetic models. A total of 1447 patients were enrolled, and 3-month follow-up data were obtained from 1388 (95.92%). Multivariate regression analysis showed that SNP87 of *PDE4D* gene was associated with increased risk of unfavorable outcome after total ischemic stroke (*OR* = 1.47, 95%*CI* 1.12–1.93), as well as stroke due to large-artery atherosclerosis (*OR* = 1.49, 95%*CI* 1.04–2.11) and small-artery occlusion (*OR* = 1.76, 95%*CI* 1.05–2.96) under a recessive model. No association between SNP83 genotype and poor outcome was found. Overall, this study demonstrated that the *TT* genotype of SNP87 in *PDE4D* was associated with increased risk of poor outcome after total ischemic stroke, large-artery atherosclerosis and small-artery occlusion, in a Chinese population.

Stroke is the second most common cause of death worldwide and the first leading cause of mortality in China, with an annual mortality of approximately 1.6 million^1^,^2^,^3^. It has different subtypes, and ischemic stroke (IS) accounts for about 85%^4^. Traditional risk factors for ischemic stroke such as hypertension, dyslipidemia, diabetes mellitus, and smoking are well-established, but not fully known^5^,^6^. Various epidemiological studies from twins, case-control and cohort studies of familial aggregation suggest that genetic predisposition may contribute to IS, and it is more of a clinical syndrome rather than a disease due to numerous clinical, genetic, and lifestyle risk factors^7^,^8^.

One widely studied candidate gene is phosphodiesterase 4D (*PDE4D*), which was identified via genome-wide association studies (GWAS). *PDE4D* is located on chromosome 5q12 that spans a 1.6-Mb region and contains 24 exons, 8 splice variants and hundreds of gene variants. It belongs to a superfamily of phosphodiesterases (PDE4 family), and encodes cyclic adenosine monophosphate (cAMP) -specific 3′,5′-cyclic phosphodiesterase 4D, which plays an important role in the degradation of cAMP^9^. The proliferation and migration of vascular smooth muscle cells and macrophages is responsible for atherosclerosis, and cAMP is involved in this process^10^. In 2003, Gretarsdottir *et al*. first identified the association of SNP83 in the *PDE4D* gene with carotid stroke in an Icelandic population^11^. Since then, many studies have found such association in various populations^12^. Woo *et al*. found that *PDE4D* was associated with ischemic stroke and, in particular, with cardio-embolic stroke among whites and blacks^13^. Milton *et al*. conducted a cohort study in Australia, and also detected a positive association of *PDE4D* and cardio-embolic stroke^14^. No association was found between *PDE4D* and ischemic stroke in German and Chinese populations^15^,^16^. These inconsistent results may be due to different allele frequencies across study populations, particularly in different ethnicities and geographical groups. In addition, several meta-analyses suggested that *PDE4D* was related to ischemic stroke, especially in Asian and Chinese populations^17^,^18^,^19^.

A few studies explored the association of genetic polymorphism and outcome after ischemic stroke^20^,^21^,^22^,^23^. However, to our knowledge, there are still no studies reporting the association of *PDE4D* genetics with prognosis. Ischemic stroke has been classified into different subtypes due to different pathogenesis^24^. Therefore, in this study we aimed to investigate the association of *PDE4D* genetics with 3-month outcome after ischemic stroke, and its different subtypes classified by the Trial of Org10172 in Acute Stroke Treatment (TOAST) classification in a Chinese population.

## Results

### Clinical characteristics

A total of 1447 ischemic stroke patients were enrolled in the study, and 59 patients (4.08%) without 3-month data were excluded. The mean age of the included patients was (60.04 ± 3.01) years, and the male patients accounted for 75.1%. The baseline characteristics of patients are presented in Table 1.

Compared with patients presenting a favorable outcome, patients with unfavorable outcome were older (62.25 ± 2.26 *vs.* 55.98 ± 3.39 years, *P* < 0.001), with a significantly higher proportion of women (27.3% *vs.* 20.6%, *P* < 0.05), less history of hyperlipidemia (14.3% *vs.* 19.4%, *P* < 0.05), more history of ischemic stroke (27.3% *vs.* 18.6%, *P* < 0.001), and higher NIHSS on admission [6(2–10) *vs.* 3(1–5), *P* < 0.001]. Differences in other demographics and medical history were not significant between the two groups. According to TOAST classification, the proportion of large-artery atherosclerosis was higher in the unfavorable outcome group (63.5% *vs.* 54.9%, *P* < 0.05).

### Distributions of SNP83 and SNP87 genotypes

Distributions of the SNP83 and SNP87 genotypes are shown in Table 2. Genotype distributions of SNP87 (rs918592) and SNP83 (rs966221) both followed the Hardy-Weinberg equilibrium (χ^2^ = 0.304, *P*_HWE_ = 0.582; χ^2^ = 0.656, *P*_HWE_ = 0.418, respectively). The minor allele frequencies (MAF) of rs918592 and rs966221 were 0.472 and 0.215, respectively.

### Associations of SNP83 and SNP87 with 3-month outcome after ischemic stroke and different subtypes

Univariate logistic regression analysis showed significant association between SNP87 genotype and unfavorable outcome after ischemic stroke under the additive model (TT *vs.* CT *vs.* CC) (*OR* 1.19, 95%*CI* 1.01–1.39) and recessive model (TT *vs.* CT + CC) (*OR* 1.47, 95%*CI* 1.14–1.89). After adjustment for age, gender and NIHSS on admission, multivariate logistic regression analysis revealed that the association remained significant under the additive (*OR* 1.19, 95%CI 1.01–1.40) and recessive (*OR* 1.47, 95%*CI* 1.12–1.93) models (Table 3). However, no association was found between the SNP83 genotype and unfavorable outcome after ischemic stroke.

In subgroup analysis based on the TOAST classification, we found significant association between SNP87 genotype and unfavorable outcome after stroke due to large-artery atherosclerosis (*OR* 1.49, 95%*CI* 1.04–2.11) and small-artery occlusion (*OR* 1.76, 95%*CI* 1.05–2.96) under a recessive model (Table 4) after adjustment for age, gender and NIHSS on admission. However, no association was found between the SNP83 genotype and unfavorable outcome in any stroke subtype.

## Discussion

In this study, we found that the TT genotype of SNP87 in *PDE4D* was associated with an increased risk for 3-month unfavorable outcome after total ischemic stroke, as well as stroke due to large-artery atherosclerosis and small-artery occlusion, in a Chinese population. However, no association was found between the SNP83 genotype and unfavorable outcome after ischemic stroke or any subtype.

After Gretarsdottir *et al*. identified *PDE4D* as a susceptible gene for ischemic stroke^11^, a large number of studies explored the association of single nucleotide polymorphisms across this gene with the disease. *PDE4D* is located in chromosome 5q12, and encodes cyclic adenosine monophosphate (cAMP) -specific 3′,5′-cyclic phosphodiesterase 4D^8^. *PDE4D* selectively degrades the second messenger cAMP in vascular smooth muscle cells and activates macrophages, which is a key signaling molecule mediating proliferation, migration and secretion of cells related to atherosclerosis and plaque stability. Thus, *PDE4D* has been shown to contribute to ischemic stroke via the atherosclerotic pathway^10^,^25^,^26^,^27^.

Due to varying allele frequencies among different populations, ethnicities and geographies, several studies focused on the association of *PDE4D* with ischemic stroke, obtaining conflicting results, even in Chinese populations^13^,^14^,^15^,^16^,^28^,^29^,^30^,^31^,^32^. He *et al*. demonstrated a strong association of rs918592 in the PDE4D gene with ischemic stroke in the Henan Han population, and also suggested that rs918592 and rs2910829 polymorphisms were associated with ischemic stroke in young Chinese individuals^31^,^32^. Shao *et al*. found no association of PDE4D polymorphisms with ischemic stroke in a southeastern Chinese population^15^. Liu *et al*. conducted a meta-analysis of Chinese population studies, and showed a positive correlation between rs2910829 and ischemic stroke^18^. In the present study, we evaluated the association of *PDE4D* with 3-month outcome after ischemic stroke in a Chinese population. In this cohort, the patients were relatively young with a mean age of about 60 years, which was slightly younger compared with the patients in the China National Stroke Registry (mean age of about 63.8 years)^33^. We found that the SNP87 genotype in *PDE4D* was associated with an increased risk of 3-month unfavorable outcome after ischemic stroke under the additive and recessive models. Further, multivariate analysis showed that this association was still significant under the additive and recessive models after adjustment for age, gender, NIHSS on admission and even other clinical characteristics. The results were consistent with the findings from previous studies on *PDE4D* genetics with the incidence of ischemic stroke among Chinese populations^18^,^31^,^32^. However, no association was found between SNP83 genotype in *PDE4D* and unfavorable outcome after ischemic stroke, which may be explained by the relatively small sample size.

Ischemic stroke is a complicated disease and is dividedinto five subtypes according to TOAST classification including large-artery atherosclerosis, cardio-embolism, small-artery occlusion, stroke of other determined etiology and stroke of undetermined etiology^34^. As the subtypes have different pathogeneses, genetic polymorphisms may be susceptible to specific subtype. In this study, we performed a subgroup analysis by stroke subtype, and found that SNP87 was associated with an increased risk of unfavorable outcome after stroke due to large-artery atherosclerosis and small-artery occlusion, under a recessive model. This result can be explained by the role of *PDE4D* in atherosclerosis, as mentioned above. Usually, cardio-embolism is associated with particularly poor outcome^35^. However, we did not find such association in the current study. This may be due to the relatively small sample size of the subtype caused by cardio-embolism. Therefore, additional studies with larger sample are needed to demonstrate this association.

Our study has a few advantages. First, ischemic stroke manifests different pathogenesis, and the *PDE4D* gene may be associated with a specific subtype. Therefore, we conducted subgroup analysis according to the TOAST classification. Further, IS subtype was classified by two experienced neurologists, which improved the accuracy of IS subtype classification. Finally, with more comprehensive etiological investigations (e.g. most patients performed imaging examination), IS subtype classification was more precise, and more patients were classified into other subtypes, thus leading to a lower proportion of undetermined etiology.

However, several potential limitations exist in the present study. First, we only selected patients from Beijing Tiantan Hospital, which may not be representative of all patients in China, and might lead to a bias. Therefore, the percentage of GG genotype of SNP83 was only 4.3%, which may be underpowered to detect the effect. Second, we only focused on SNP87 and SNP83 genetic polymorphisms in *PDE4D*, not assessing other genes or environmental factors. The potential role of *PDE4D* genetic polymorphisms may be reduced or absent in other gene-gene or gene-environment interactions. In addition, sample size was relatively small. Therefore, additional studies in other populations, with larger sample size, are needed to demonstrate the association of genetic polymorphisms with IS susceptibility.

## Methods

### Study population

Ischemic stroke patients were consecutively enrolled at the Beijing Tiantan Hospital from October 2009 to December 2013. IS was defined as a loss of global or focal cerebral function persisting for >24 hours, with corresponding infarction on brain imaging and a probable vascular cause excluding non-vascular causes (such as primary and metastatic neoplasms, post-seizure paralysis, or head trauma), and intracerebral hemorrhage on computed tomography (CT) or magnetic resonance imaging (MRI)^36^. Inclusion criteria were: (1) >18 years old; (2) acute ischemic stroke within 14 days; (3) patients undergoing diffusion weighted imaging (DWI) of the brain; blood samples drawn within 48 hours for DNA extraction. Patients diagnosed with silent cerebral infarction, transient ischemic attack (TIA), intracerebral hemorrhage (ICH) or subarachnoid hemorrhage (SAH) were excluded.

This study was approved by the Ethics Committee of Beijing Tiantan Hospital, Capital Medical University (Approval No. 2009-005). All experiments were conducted in compliance with national guidelines, following the Ethics and methods for biological rhythm research on animals and humans^37^. Written informed consent was obtained from each patient or legal proxy.

### Baseline data collection and follow-up

Clinical data were collected, including demographics, medical history, family history, pre-stroke modified Rankin Scale (mRS), National Institutes of Health Stroke Scale (NIHSS) on admission, physical and neurological examinations, laboratory data, electrocardiogram (ECG), ultrasonic cardiogram (UCG), carotid artery ultrasound findings, transcranial Doppler, brain CT and/or MRI examinations, and high-resolution MRI or transesophageal echocardiography (TEE) if available. The stroke subtype of each patient according to the TOAST classification^34^ was identified independently by two experienced neurologists (Y.L.S. and C.J.W.) in Beijing Tiantan Hospital before patient discharged from the hospital. Patients with large-artery atherosclerosis should have clinical and brain imaging findings of either significant (>50%) stenosis or occlusion of intra-/extracranial criminal artery, presumably due to atherosclerosis. The stenosis evaluation of intracranial supplying arteries were assessed by computed tomography angiography (CTA) or magnetic resonance angiography (MRA) or digital subtraction angiography (DSA) and that of extracranial supplying arteries were assessed by carotid ultrasonography, carotid CTA, contrast-enhanced MR angiography (CEMRA) or DSA. Both neurologists were blinded to the follow-up data and genotype information. All patients were followed up by telephone at 3 months after IS onset by trained interviewers. The interviewers were blinded to patient’s clinical symptoms and genotypes. Unfavorable outcome was defined as modified Rankin Scale (mRS) ≥ 2^38^.

### *PDE4D* genotypin

DNA was extracted from peripheral white blood cells by the phenol–chloroform method^39^. The SNP83 (rs966221) and SNP87 (rs918592) genotypes in *PDE4D* were determined by polymerase chain reaction (PCR). PDE4D genotyping of the included population was performed by time-of-flight mass spectrometry on a MASSarray platform equipped with the iPLEX genotyping technology.

### Statistical analysis

Patient characteristics were presented as mean ± standard deviation (SD) for continuous variables, or absolute count and percentage for categorical ones. The χ^2^-test and Student’s t test were used to compare categorical and continuous variables, respectively. The χ^2^-test was also used to assess the Hardy-Weinberg equilibrium (HWE). Odds ratios (ORs) and 95% confidence intervals (CIs) were calculated to assess the associations of PDE4D genetic polymorphisms with outcome after ischemic stroke and its different subtypes under additive, dominant and recessive genetic models. Unfavorable outcome was defined as modified Rankin Scale (mRS) ≥ 2^38^. For SNP87, the additive model was TT *vs.* CT *vs.* CC; dominant and recessive models were TT + CT *vs.* CC and TT *vs.* CT + CC, respectively. For SNP 83, additive, dominant, and recessive models were GG *vs.* AG *vs.* AA, GG + AG *vs.* AA, and GG *vs.* AG + AA, respectively. Sample size was calculated with PASS software version 11.0, and the sample size required was 1277 with the assumption that the minor allele frequency was 20%, Odds Ratio was 1.5, power was 0.8, and alpha was 10^−7^. *P* < 0.05 was considered statistically significant. Statistical analyses were performed with the SAS software version 9.4 (SAS Institute Inc., Cary, NC).

## Additional Information

**How to cite this article**: Song, Y.- *et al*. Phosphodiesterase 4D polymorphisms associate with the short-term outcome in ischemic stroke. *Sci. Rep.* **7**, 42914; doi: 10.1038/srep42914 (2017).

**Publisher's note:** Springer Nature remains neutral with regard to jurisdictional claims in published maps and institutional affiliations.

## Figures and Tables

**Table 1:** Baseline characteristics according to the subgroup outcomes.

Variables	Total	Outcome		*P*
		Unfavorable (mRS < 2)	Favorable (mRS ≥ 2)	
N	1388 (100.0)	490 (35.3)	898 (64.7)	
Age(mean ± SD)	60.04 ± 3.01	55.98 ± 3.39	62.25 ± 2.26	<0.001
Gender[*n*(%)]				
Male	1042 (75.1)	389 (79.4)	653 (72.7)	0.006
Female	346 (24.9)	101 (20.6)	245 (27.3)	
Ethnic[*n*(%)]				
Han	1356 (97.7)	479 (97.8)	877 (97.7)	0.912
Other	32 (2.3)	11 (2.2)	21 (2.3)	
Education[*n*(%)]				
Primary school or lower	805 (58.0)	258 (52.7)	547 (60.9)	0.003
Senior school or higher	583 (42.0)	232 (47.3)	351 (39.1)	
History[*n*(%)]				
Smoking	821 (60.5)	306 (63.4)	515 (58.9)	0.110
Drinking	315 (25.3)	120 (26.4)	195 (24.6)	0.479
Hypertension	853 (61.5)	287 (58.6)	566 (63.0)	0.103
Diabetes	400 (28.8)	130 (26.5)	270 (30.1)	0.165
Hyperlipidemia	223 (16.1)	95 (19.4)	128 (14.3)	0.013
Coronary heart disease	142 (10.2)	46 (9.4)	96 (10.7)	0.444
Atrial fibrillation	52 (3.7)	12 (2.4)	40 (4.5)	0.060
Ischemic stroke	336 (24.2)	91 (18.6)	245 (27.3)	<0.001
Cerebral hemorrhage	32 (2.3)	12 (2.4)	20 (2.2)	0.792
NIHSS on admission	4 (2–8)	3 (1–5)	6 (2–10)	<0.001
TOAST classification				
Large-artery atherosclerosis	839 (60.5)	269 (54.9)	570 (63.5)	0.006
Small-artery occlusion	76 (5.5)	24 (4.9)	52 (5.8)	
Cardio-embolism	321 (23.1)	132 (26.9)	189 (21.1)	
Other determined etiology	28 (2.0)	15 (3.1)	13 (1.5)	
Undetermined etiology	124 (8.9)	50 (10.2)	74 (8.2)	

Note: Continuous and categorical variables were tested by Student’s t-test and χ^2^ analysis, respectively.

NIHSS: national institutes of health stroke scale.

**Table 2:** Genotype of SNP 83 and SNP87 in PDE4D gene between different groups.

Genotype	Total[*n*(%)]	Outcome[*n*(%)]		MAF	*P*_HWE_
		Favorable	Unfavorable		
SNP87(rs918592)					
CC	302 (21.9)	110 (22.5)	192 (21.5)	0.472	0.582
CT	698 (50.6)	267 (54.7)	431 (48.3)		
TT	380 (27.5)	111 (22.8)	269 (30.2)		
SNP83(rs966221)					
AA	845 (61.2)	300 (61.6)	545 (61.0)	0.215	0.418
AG	477 (34.5)	165 (33.9)	312 (34.9)		
GG	59 (4.3)	22 (4.5)	37 (4.1)		

Note: SNP, single nucleotide polymorphism; MAF, minor allell frequency; HWE, Hardy-Weinberg equilibrium.

**Table 3:** Association between PDE4D genotype and outcome after ischemic stroke.

Genetic model	*OR*	95%*CI*	Adjusted *OR*†	95%*CI*	Adjusted *OR*‡	95%*CI*
SNP87						
Additive model	**1.19**	**1.01**–**1.39**	**1.19**	**1.01**–**1.40**	**1.18**	**1.00**–**1.39**
Recessive model	**1.47**	**1.14**–**1.89**	**1.47**	**1.12**–**1.93**	**1.47**	**1.12**–**1.93**
Dominant model	1.06	0.81–1.38	1.06	0.80–1.40	1.03	0.78–1.37
SNP83						
Additive model	1.01	0.83–1.22	1.01	0.83–1.24	1.02	0.84–1.26
Recessive model	0.93	0.54–1.59	0.92	0.52–1.63	0.91	0.51–1.61
Dominant model	1.02	0.81–1.28	1.03	0.81–1.31	1.05	0.83–1.34

Note: ^†^adjustment for age, gender and NIHSS on admission; ^‡^adjustment for age, gender, education, history of hypertension, diabetes, hyperlipidemia, atrial fibrillation, ischemic stroke, NIHSS on admission.

**Table 4:** Association between PDE4D genotype and outcome after ischemic stroke subtypes.

Stroke subtype	Additive model		Recessive model		Dominant model	
	*OR*	95%*CI*	*OR*	95%*CI*	*OR*	95%*CI*
SNP87						
Large–artery atherosclerosis	1.20	0.97–1.49	**1.49**	**1.04**–**2.11**	1.08	0.75–1.56
Cardio-embolism	0.86	0.38–1.93	1.26	0.34–4.66	0.49	0.12–2.07
Small-artery occlusion	1.37	0.99–1.89	**1.76**	**1.05**–**2.96**	1.30	0.75–2.25
SNP83						
Large-artery atherosclerosis	0.90	0.69–1.16	0.68	0.34–1.34	0.92	0.68–1.26
Cardio-embolism	1.17	0.48–2.82	1.00	0.13–7.89	1.30	0.41–4.06
Small-artery occlusion	1.20	0.79–1.82	2.15	0.52–8.85	1.15	0.71–1.85

Note: adjustment for age, gender and NIHSS on admission.
